# Adherence to dietary guidelines for the Spanish population and risk of overweight/obesity in the SUN cohort

**DOI:** 10.1371/journal.pone.0226565

**Published:** 2019-12-31

**Authors:** Clara Gómez-Donoso, Miguel Ángel Martínez-González, J. Alfredo Martínez, Carmen Sayón-Orea, Carmen de la Fuente-Arrillaga, Maira Bes-Rastrollo

**Affiliations:** 1 Department of Preventive Medicine and Public Health, School of Medicine, University of Navarra, Pamplona, Spain; 2 Biomedical Research Centre Network on Physiopathology of Obesity and Nutrition (CIBERobn), Institute of Health Carlos III, Madrid, Spain; 3 Navarra Institute for Health Research (IdiSNA), Pamplona, Spain; 4 Department of Nutrition, Harvard T.H. Chan School of Public Health, Boston, MA, United States of America; 5 Department of Nutrition, Food Sciences and Physiology, School of Pharmacy and Nutrition, University of Navarra, Pamplona, Spain; University of British Columbia, CANADA

## Abstract

Dietary guidelines play a key role in setting standards for nutrition policies and promoting healthy eating. Like other public health guidelines, they are often influenced by political and economic factors that could place other concerns ahead of the population’s health. In order to determine their effectiveness on obesity prevention, we prospectively examined the association between adherence to the latest available national dietary guidelines and the incidence of overweight/obesity in a Spanish cohort study. A sample of 11,554 participants of the “Seguimiento Universidad de Navarra” (SUN) cohort, initially free of overweight or obesity, was included in the study. The Spanish Society of Community Nutrition (SENC) food pyramid (FP) score was computed based on the ratio of consumed to recommended daily servings of grains, fruits, vegetables, dairy, protein-rich foods, olive oil, red and processed meat, sweets, salty snacks and spreadable fats, fermented alcoholic beverages and water. The same approach was followed to calculate the SENC hydration pyramid (HP) score, considering the intake of water and different kind of beverages. Adherence was calculated at baseline and after 10 years of follow-up. Cox proportional hazards models were used to assess the incidence of overweight/obesity (BMI ≥25 kg/m^2^). During a median follow-up of 10.3 years, 2320 incident cases were identified. The highest level of adherence to the SENC FP score was modestly associated with a reduced risk of overweight/obesity (multivariable-adjusted HR for the fifth quintile vs. the first quintile = 0.78; 95% CI: 0.67–0.91; p-trend: 0.007). No consistent trends were found for the SENC HP. In a large prospective cohort of Spanish university graduates, we found an inverse linear association between adherence to the SENC FP and overweight/obesity risk, whereas this was not the case for the HP.

## Introduction

Chronic or non-communicable diseases (NCDs) account for 71% (41 million) of global deaths [1]. Reducing NCD mortality by 2030 is one of the challenging targets of the Sustainable Development Goals [2]. Dietary habits and nutritional status, including overweight and obesity, constitute one of the leading modifiable risk factors for NCDs. In this context, dietary guidelines are issued in order to promote overall health and delay the onset of these preventable chronic diseases. Dietary guidelines provide evidence-based nutrition recommendations that play an important role in setting nutritional public health policies and educating the population about healthy food choices. Thus, it seems important to evaluate the actual health impact of dietary guidelines in the long-term using prospective cohorts.

In contrast to other countries, neither the Spanish Ministry of Health nor the Spanish Ministry of Agriculture have developed national dietary guidelines. The first comprehensive dietary guidelines for the Spanish population were published in 1995 by experts in nutrition and public health affiliated with the Spanish Society of Community Nutrition (“Sociedad Española de Nutrición Comunitaria”, SENC, http://www.nutricioncomunitaria.org) [3]. They were updated in 2002, 2004 and, more recently, in 2016 [4–6]. Additionally, in 2018, SENC and other scientific societies of primary healthcare professionals developed a more practical version of the guidelines, with an emphasis on food sustainability. This edition was published by Editorial Planeta with the title “Healthy Dietary Guidelines for primary healthcare and citizen groups”, and its rationale has been explained in a scientific publication [7].

The latest version of the guidelines highlight the importance of physical activity, emotional status, energy balance, healthy cooking procedures and adequate hydration [6]. Beyond those new elements, recommendations have always promoted a balanced, varied and moderate diet [3–6]. However, guidelines do not provide any quantitative guidance for moderate or occasional consumption, and contextual factors are poorly aligned with moderate eating [8]. In addition, currently available evidence suggests that greater dietary diversity (i.e., eating “a varied diet” or “everything in moderation”) is not necessarily beneficial in terms of promoting an optimal body weight and healthy eating patterns [9,10].

The SENC dietary guidelines also include the possibility of a moderate and responsible consumption of fermented alcoholic beverages [6], which is inconsistent with the positioning of the Spanish Society of Epidemiology (Sociedad Española de Epidemiología; SEE) [11] and other scientific evidence in the Spanish population [12–14]. Furthermore, there is a global and national concern regarding the potential presence of commercial bias in nutrition research and an undue influence in the elaboration of dietary guidelines [15–23].

Some studies have previously examined the cross-sectional association between adherence to the SENC recommendations and body mass index (BMI), suggesting that the Spanish dietary guidelines may be an effective tool for obesity prevention [24,25]. However, no study has yet prospectively assessed the risk of developing overweight and obesity according to adherence to the latest SENC guidelines, taking into account both the food and hydration pyramid recommendations. Therefore, our aim was to evaluate this potential association in the University of Navarra Follow-up (“Seguimiento Universidad de Navarra”, SUN) Project, a large Mediterranean prospective cohort of Spanish university graduates.

## Materials and methods

### Study population

The SUN project is a dynamic (i.e., recruitment permanently open), multipurpose prospective cohort study of Spanish university graduates focused on evaluating the effects of healthy dietary patterns on the incidence of major chronic diseases. The recruitment started in December 1999 at the University of Navarra, and graduates from this and other Spanish universities are invited to participate on an annual basis. Once participants accept to enter the study, they receive a detailed questionnaire by ordinary mail or an email with a personal code to answer the questionnaire at the SUN website. Voluntary completion of the first self-administrated questionnaire is considered to imply informed consent. Every two years, shorter follow-up questionnaires are sent by ordinary mail or emailed to track changes in lifestyle habits, diagnosis of new diseases, and general well-being. The overall follow-up rate approaches 91%. Further details of its design, methods, objectives and main results to date have been published elsewhere [26]. The Institutional Review Board of the University of Navarra approved the study protocol, which was in accordance with Declaration of Helsinki guidelines. This study was registered at clinicaltrials.gov as NCT02669602.

**Fig 1:**
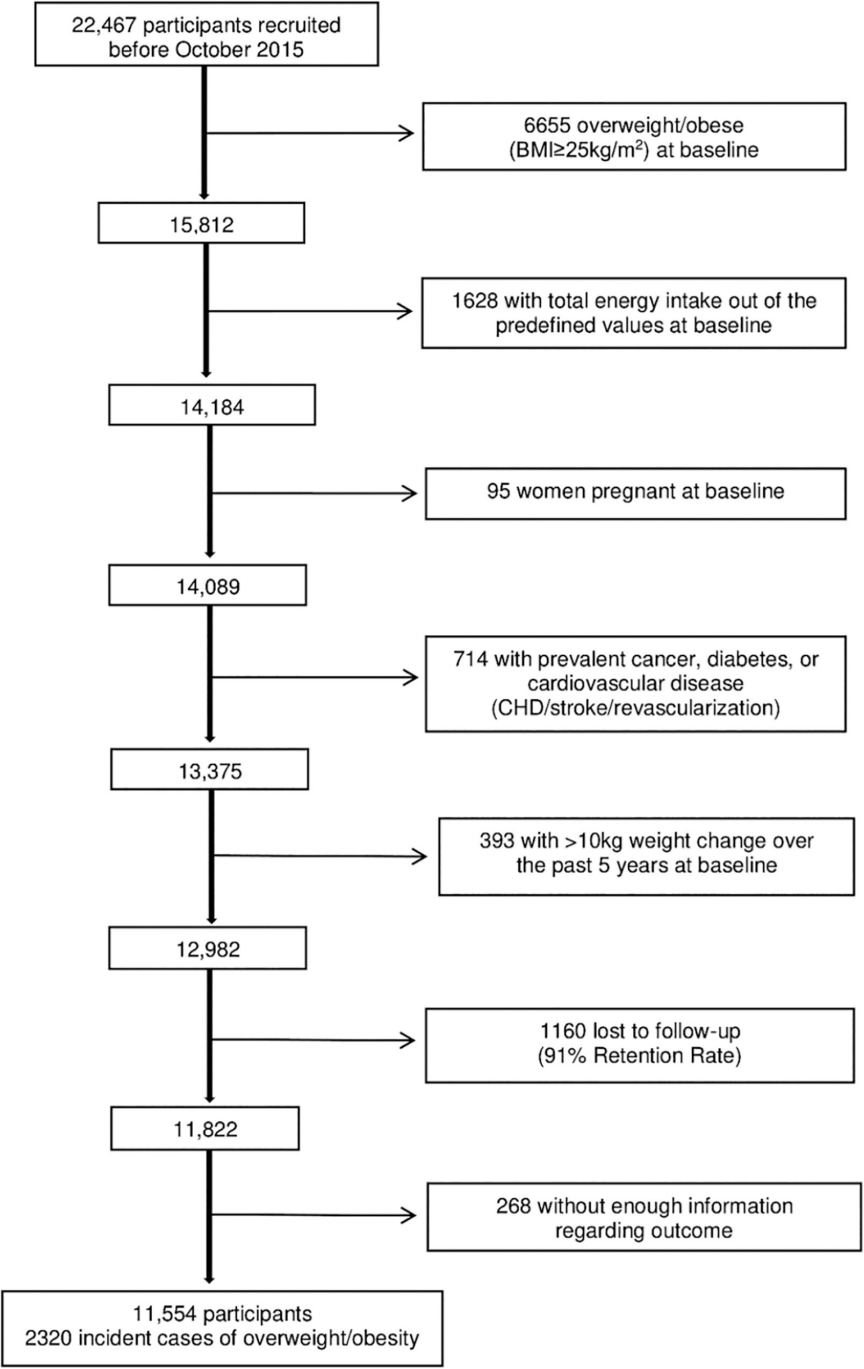
Flow chart of the selection process among participants of the SUN cohort.

Up to July 2018, the data set of the SUN project included 22,791 participants. To ensure that all participants had the opportunity to answer the 2-year follow-up questionnaire, 22,467 participants who had answered the baseline questionnaire before October 2015 were considered eligible (Fig 1)

For the present longitudinal analyses, we excluded participants with prevalent overweight or obesity (BMI ≥25 kg/m^2^) at baseline (n = 6655) and those who reported implausible values for total energy intake (n = 1628) according to predefined limits (>4000 kcal/d in men and >3500 kcal/d in women or <800 kcal/d in men and <500 kcal/d in women) in order to reduce information bias [27]. We also excluded women who were pregnant at baseline (n = 95), participants with chronic disease at baseline (diabetes, cardiovascular disease and/or cancer) (n = 714), and those with a weight change >10 kg in the previous 5 years before entering the cohort to reduce potential sources of confounding by other causes of weight change (n = 393). Among the remaining 12982 participants, 1160 were lost to follow-up, leaving a total of 11822 (retention rate = 91%) available for analysis. Additionally, we excluded 268 participants who had missing values in the variables of interest. Finally, a sample of 11,554 participants was available for the analysis.

### Exposure assessment and the SENC FP/HP score

A 136-item semi-quantitative food frequency questionnaire (FFQ) repeatedly validated in Spain [28,29] was completed at baseline and after 10 years of follow-up. The FFQ assessed participants’ usual intake of foods and beverages consumed in the past year. A typical Spanish portion size was specified for each item, and consumption frequencies were divided into 9 categories ranging from never/almost never to >6 servings/day. The nutrient databank was updated using Spanish food composition tables [30].

Adherence to the Spanish dietary guidelines was assessed according to the latest version published by the SENC committee of experts in 2016 [6,7], which included both a food pyramid (FP) and a hydration pyramid (HP) with consumption recommendations for the following food and beverage groups: grains and byproducts, fruits, vegetables, dairy, protein-rich foods, olive oil, red and processed meat, sweets, salty snacks and spreadable fats, fermented alcoholic beverages (wine and beer), water and other beverages (including coffee, tea, fruit and vegetable juices, and diet and regular soda). Physical activity recommendations (60 minutes per day) were used to estimate the daily recommended intake of grains and byproducts, as advised by the guidelines.

Dietary intakes from the baseline FFQ were used to calculate both the SENC FP and HP scores, which were updated with the use of the 10-year follow-up dietary assessment if the participant was followed for longer than 10 years and had completed the 10-y follow-up FFQ. The baseline score was used for participants that had not completed the 10-y FFQ (i.e., the last observations were carried forward) to prevent participants being excluded from the analyses. The percentage of participants who completed two FFQs was 38% (4407 out of 11,554 participants) due to the different recruiting times, according to the dynamic design (i.e., recruitment is permanently open, and the subset of participants recruited less than 10 years ago did not have the opportunity to complete the 10-y FFQ). The percentage of participants that were followed for longer than 10 years but had not completed the 10-y follow-up FFQ was 37% (2564 out of 6971).

Adherence was assessed following the method that was proposed for the German Food Pyramid Index [31], which was adapted to the components and recommendations of the SENC dietary guidelines. The details on the components included in the scores and the scoring criteria can be found in Tables 1 and 2. Briefly, the score for food groups recommended on a daily basis was calculated according to the following criteria:

- **Intakes below the recommended servings**: points assigned were given by the following Eq (1):
  $$score=\frac{servings\;consumed}{recommended\;servings\;(lower\;limit)}\times 10$$
- **Intakes within the recommended servings**: 10 points were assigned.
- **Intakes exceeding the recommended servings**: points assigned were given by the following Eq (2):
  $$score=\frac{recommended\;servings\;(upper\;limit)}{servings\;consumed}\times 10$$

Points assigned were different in the case of fruits, vegetables and water because recommended servings refer to a minimum intake, not maximum.

○ Fruits and vegetables: 20 points were assigned (i.e., 10 extra points were assigned if recommendations were exceeded to account for potential health benefits of intakes above the recommended).
○ Water: 10 points were assigned.

In addition, 10 extra points were given if whole-grain bread was preferred (the SENC FP recommends to eat all grains as whole grains, but information on other whole-grain products was unavailable in our cohort) and protein food sources were varied (i.e., lean meat, fish and shellfish, eggs, legumes and nuts).

**Table 1:** Scoring criteria: SENC Food Pyramid (2016).

Food groups and FFQ items included		*RS*	Scoring		
			Criteria	Value (points)	Range (points)
	***Recommendation*: *On a daily basis***				
	**Cereal grains and byproducts** Pasta, rice, cereals, bread, potato, green peas	4, 5 or 6^c^	*S* ≤*RS*	Eq.(1)^a^	0–20
			*S* >*RS*	Eq.(2)^b^	
			Whole-grain bread^d^	10	
	**Fruits** Citrus, grapes, banana, apple, pear, strawberry, peach, apricot, nectarine, cherries, plums, figs, melon, watermelon, mango, papaya, kiwi, fruit canned in its own juice, dried fruit^e^, 100% fruit juice^f^	3–4	*S* <3	Eq.(1)	0–20
			*S* ≥3 and *S* ≤4	10	
			*S* >4	20	
	**Vegetables** Swiss chard, spinach, cabbage, cauliflower, broccoli, lettuce, chicory, carrot, tomatoes, green beans, eggplant, zucchini, cucumber, pepper, asparagus, others	2–3	*S* <2	Eq.(1)	0–20
			*S* ≥2 and *S* ≤3	10	
			*S* >3	20	
	**Dairy** Milk, milk-based products, yogurt, fresh cheese	2–3	*S* <2	Eq.(1)	0–10
			*S* ≥2 and *S* ≤3	10	
			*S* >3	Eq.(2)	
	**Protein-rich foods** Lean meat (chicken, turkey, rabbit), fish & shellfish, eggs, legumes, nuts	1–3	*S* <1	Eq.(1)	0–20
			*S* ≥1 and *S* ≤3	10	
			*S* >3	Eq.(2)	
			Varied sources^g^	10	
	**Olive oil**	4.5–5.5	*S* <4.5	Eq.(1)	0–10
			*S* ≥4.5 and *S* ≤5.5	10	
			*S* >5.5	Eq.(2)	
	**Water**	4–6	S <4	Eq.(1)	0–10
			S ≥4	10	
	***Recommendation*: *Optional*, *occasional and moderate***^***h***^				
	**Red and processed meats** Beef, veal, pork, lamb, liver, other viscera, serrano ham, cooked ham, spicy pork sausage, salami, mortadella, foie-gras, black pudding, bacon, other cured or smoked meats, hamburger, hot dog	<1	*S* <1	10	0–10
			S ≥1	Eq.(2)	
	**Sweets, salty snacks and spreadable fats** Custard, pudding, ice-cream, potato chips, cookies, muffins, donuts, croissant, cakes, churros, chocolates, nougat, marzipan, ready-to-consume pies and other formulations, instant soups, pizza, margarine, mayonnaise	<1	*S* <1	10	0–10
			*S* ≥1	Eq.(2)	
	**Fermented alcoholic beverages** Wine and beer	1 or 2^f^	*S* ≤*RS*	10	0–10
			*S* >*RS*	0	

^a^ Eq (1):
$$score=\frac{servings\;consumed\;(S)}{recommended\;servings\;(RS)\;lower\;limit}\times 10$$

^b^ Eq (2):
$$score=\frac{recommended\;servings\;(RS)\;upper\;limit}{servings\;consumed\;(S)}\times 10$$

^c^ According to daily physical activity (PA): *RS* = 4 if PA <30 min/day; *RS* = 5 if PA ≥30 and PA ≤60 min/d; *RS* = 6 if PA >60 min/d.

^d^ 10 extra points were given if at least 0.5 *S* of whole-grain bread were consumed.

^e^ Dried fruit (raisins, figs) were included within recommended fruit intake if fresh fruit intake ≥3 *S*.

^f^ 100% fruit juices were included within recommended fruit intake if juice intake ≤1 *S*.

^g^ 10 extra points were given if participants varied their protein sources (≥3 different ones per day).

^h^ “Optional, occasional or moderate” consumption was defined as <1 *S* based on previous research [25].

^f^ According to sex: *RS* = 1 for women; *RS* = 2 for men.

**Table 2:** Scoring criteria: SENC Hydration Pyramid (2016).

Beverage groups and FFQ items included			*RS*	Scoring		
				Criteria	Value (points)	Range (points)
	***Recommendation*: *On a daily basis***					
	Level 1	**Water** Tap and bottled mineral water	10^c^	*S* <10	Eq.(1)^a^	0–20
	Level 2	**Low/non-caloric beverages**^**d**^ Diet (artificially sweetened) carbonated beverages and non-sugared coffee		*S* = 10	10	
	Level 3	**Caloric beverages with some nutrients**^**e**^ Vegetable juices (i.e., ‘gazpacho’), broths, fresh fruit juices, milk and milk-based products, sugared coffee^f^		*S* >10	Eq.(2)^b^	
				Compliance with the hierarchy levels^g^	10	
	***Recommendation*: *Optional*, *occasional and moderate***^***h***^					
	Level 4	**Calorically sweetened beverages without nutrients** Sugar-sweetened beverages and commercial fruit juices	1	*S* <1	10	0–10
				S ≥1	Eq.(2)	

^a^ Eq (1):
$$score=\frac{servings\;consumed\;(S)}{recommended\;servings\;(RS)}\times 10$$

^b^ Eq (2):
$$score=\frac{recommended\;servings\;(RS)}{servings\;consumed\;(S)}\times 10$$

^c^ Guidelines recommend to consume a total of 10 servings per day of water, low/non-caloric beverages, caloric and nutritive beverages, and water coming from foods.

^d^ Information on tea consumption was unavailable as it was not included in the FFQ.

^e^ Information on milk replacers, alcohol-free beer, and sports drinks consumption was not included in the FFQ. Sugar content of milk and milk-based products was unavailable.

^f^ Information on sugared coffee was unavailable, so we distinguished between consumption of coffee with or without added sugar based on the question “Do you add sugar to some beverages?”.

^g^ 10 extra points were assigned to those participants who complied with hierarchy levels of the beverage groups recommended on a daily basis (consumption of water>low/non-caloric beverages>caloric beverages)

^h^ “Optional, occasional or moderate” consumption was defined as <1 *S* based on previous research [25].

The score for food groups that are limited to an “optional, occasional and moderate” consumption was calculated using the same equations, but with different criteria. Due to their nutritional composition, these foods are not recommended on a daily basis and is preferable to eat as little as possible. Guidelines do not provide any quantitative guidance regarding these food groups, so we defined the maximum recommended frequency of occasional consumption as <1 serving per day based on previous research [25]

- **Intakes below and within the recommended servings**: 10 points were assigned.
- **Intakes exceeding the recommended servings**: points assigned were given by *Eq (2)*, with the exception of alcoholic beverages, for which 0 points were assigned to participants who exceeded the maximum intake (i.e., 1 serving per day for women and 2 for men) to account for potential health risks of intakes above the recommended.

In total, the score quantified the consumption of seven food groups recommended on a daily basis and three food groups that should only be occasionally consumed. Each of the ten components ranged from 0 (non-compliance) to 10 points (perfect compliance). Therefore, the SENC FP score ranged from 0 (lowest adherence) to 100, plus 40 extra points (highest adherence).

Likewise, the score for beverage groups recommended on a daily basis was calculated according to the following criteria:

- **Intakes below the recommended servings**: points assigned were given by *Eq (1)*.
- **Intakes within the recommended servings:** 10 points were assigned.
- **Intakes exceeding the recommended servings:** points assigned were given by *Eq (2)*.

In order to take into account the preference for water over other beverages and preference for low/non-caloric beverages over caloric beverages, 10 extra points were assigned to those participants who complied with the hierarchy levels of the HP (i.e., the first level of the HP includes the most recommended beverages and the fourth level the least recommended beverages).

The score for calorically sweetened beverages without nutrients limited to occasional intake (defined as <1 serving per day) was given by the following criteria:

- **Intakes below the recommended servings**: 10 points were assigned.
- **Intakes within and exceeding the recommended servings**: points assigned were given by *E (2)*.

Thus, the total SENC HP score ranged from 0 (lowest adherence) to 20 plus 10 extra points (highest adherence).

### Outcome assessment

Weight and height were self-reported by participants at baseline and every 2 years of follow-up. BMI was calculated as weight in kilograms divided by height in meters squared (kg/m^2^). Self-reported weight and BMI have been previously validated in a representative subsample of the SUN cohort finding highly correlated results [32]. The correlation coefficients between measured and self-reported weight and BMI were 0.991 (95% CI: 0.986–0.994) and 0.944 (95% CI: 0.986–0.965), respectively. Incident cases of overweight and obesity were defined as those participants with BMI <25 kg/m^2^ at baseline and BMI ≥25 kg/m^2^ during follow-up.

### Covariate assessment

Considering that risk of overweight/obesity is determined by multiple factors, several covariates were used for multivariable adjustment. In addition to dietary data, the baseline questionnaire gathered information on anthropometric, sociodemographic, medical and lifestyle variables (e.g., sex, age, marital status, smoking status, sleeping hours, television viewing, eating attitudes like snacking between meals and following a special diet, and family medical history). Physical activity was evaluated with the use of a previously validated questionnaire that included information such as frequency and time spent in 17 sport activities [33]. Total minutes of physical activity per day were calculated by summing up time spent in each activity. The daily physical activity of participants was quantified to estimate the recommended intake of grains and byproducts according to dietary guidelines.

### Statistical analysis

Participants were classified into quintiles (Q) of adherence to the SENC FP: Q1 (<68), Q2 (68–76), Q3 (76–83), Q4 (83–93), and Q5 (>93). Because the variability for the HP score range was limited among participants in our cohort, adherence to the SENC HP was classified in tertiles (T): T1 (<11), T2 (11–15) and T3 (>16). The use of quantiles is consistent with the fact that dietary assessment has been done using FFQs, which are tools better suited to rank individuals rather than to accurately measure absolute intakes.

Inverse probability weighting was used to determine the age- and sex-adjusted baseline characteristics of participants according to quintiles of the SENC FP and tertiles of the SENC HP in order to remove differences that were only explained by the different age and sex distribution across baseline adherence to the SENC FP and HP.

To determine the contribution of each food group to the between-person variance in adherence to the SENC FP, we constructed a series of nested least-squares linear regression models after stepwise-selection regression analyses [27]. The additional contribution of a given food group was reflected in the cumulative *R*^2^.

Cox proportional hazards models were used to evaluate whether conformity with the SENC dietary guidelines was associated with the development of overweight/obesity over the follow-up time. Hazard Ratios (HR) and their 95% Confidence Intervals (CI) were estimated considering the lowest quantile as the reference. To minimize the potential effect of diet variation during follow-up and reduce measurement error, dietary data was updated after 10 year of follow-up. The follow-up time was defined as the interval between the date of recruitment to the date of the follow-up questionnaire in which the participant entered in the category of overweight/obesity for the first time, the date of death, or the date of the last questionnaire. The Cox model included age as the underlying time variable for all analyses. After age- and sex adjusted analyses, multivariable models were additionally adjusted for known risk factors of weight gain and potential confounders (selection based on a priori subject-matter knowledge) by using baseline values of the following covariates: BMI (kg/m^2^, continuous), physical activity (METs-h/week, quartiles), hours of TV watching (quartiles), smoking status (never, current, or former), marital status (single, married, other), highest level of education achieved (graduate, postgraduate), total energy intake (kcal/day, continuous), snacking between meals (yes, no), following a special diet at baseline (yes, no), family history of obesity (yes, no) and hours of siesta sleep (0, >0 – ≤0.5, >0.5). Analyses were stratified by age groups (10-y periods) and year of recruitment (4-y periods). Robust standard errors (SEs) were used. Tests of linear trend across increasing quantiles of adherence to the SENC FP and HP were conducted for risk of overweight/obesity. The median values of adherence to the scores were assigned for each category and were used as continuous variables in the Cox models to assess dose-response relationships. We checked the proportional-hazards assumption using Schoenfeld residuals. The results for dummy variables for each quantile of the exposure were non-significant, suggesting the effect was not time-varying.

Multiple linear regression models were used to evaluate the association between quintiles of the SENC FP and average yearly weight change during follow-up. We estimated β regression coefficients and their 95% CI, which should be interpreted as the difference in average yearly weight change (g/y) for each of the upper four quintiles versus the lowest quintile.

Generalized estimating equations (GEE) were fitted to examine the association between quintiles of the cumulative average SENC FP score and average BMI during follow-up, which was biennially assessed. An exchangeable correlation structure was assumed, and the distribution was set to Gaussian and link function to identity. Absolute means were adjusted for all the potential confounders previously described in the multivariable Cox models, and extreme and median quintiles were graphically represented. In addition, to account for a potential non-linear relationship between the SENC FP score (as a continuous variable) and incident overweight/obesity, we used restricted cubic splines. The results were adjusted for the same confounding factors as the main Cox regression analysis.

Furthermore, the influence of single food groups constituting the SENC FP on incidence of overweight/obesity was examined by fitting the multivariable Cox models with each component of the score individually and adjusting for the other components.

Given that the SENC FP provides no guidance on the maximum recommended frequency of consumption of occasional food (i.e., red and processed meats, sweets, salty snacks and spreadable fats) and inclusion of moderate alcohol consumption has been contested, the simultaneous effect of varying quantitative definitions regarding the consumption of occasional food (<1 serving/day vs. <2 servings/week) and alcoholic beverages (≤1–2 servings/day vs. abstainers) was graphically represented as an ancillary analysis. The SENC FP score was treated as a dichotomous variable using the median value as the cut-off in order to represent the entire sample in the figure.

Finally, multiplicative interactions (effect modification) between adherence to the SENC FP and sex, age, physical activity (under/above the median [17 METS-h/week]) and baseline BMI (under/above the median [22 kg/m^2^]) were evaluated using likelihood ratio tests that compared the fully adjusted Cox regression model and the same model with the interaction product-term.

Sensitivity analyses were conducted to explore the robustness of our findings by refitting the multivariable-adjusted Cox regression model under several scenarios: excluding participants with energy limits between 5th and 95th percentiles, no answer in >12 items in the 136-item baseline FFQ, following a special diet at baseline, and early incident cases of overweight (participants who became overweight only after 2 years of follow-up); including participants with weight change >10kg over the past 5 years before entering the study; additionally adjusting for weight gain ≥3kg over the past 5 years before entering the cohort; without adjusting for snacking between meals and total energy intake, and considering outcome as obesity (BMI ≥ 30 kg/m^2^).

All *P* values were two-tailed, and *P* < 0.05 was considered significant. Statistical analyses were performed with the use of STATA version 12.0 software (StataCorp LP).

## Results

The study included 8419 women and 3135 men with a mean age of 35 years at baseline. We identified 2320 cases of overweight/obesity during a median follow-up period of 10.3 y (113,212 person-years). The median value of the SENC FP and HP score for the entire sample was 80 (range: 25 to 137) and 11 (range: 2 to 30), respectively.

On average, adherence to the SENC FP was higher among those participants with a family history of obesity, following a special diet, not snacking between meals, non-smokers and more physically active. Regarding nutrient intake, subjects with a higher baseline adherence to the SENC FP had a lower intake of fat (% energy) and higher intake of carbohydrates, fiber, vitamins and minerals (Table 3). Participants in the third tertile, with higher SENC HP scores, were more likely to have a lower SENC FP score and be less physically active than those in the first tertile (Table 4).

**Table 3:** Age- and sex-adjusted* baseline characteristics of participants according to quintiles (Q) of adherence to the SENC Food Pyramid (FP): The SUN Project, 1999–2015.

Variable	Adherence to SENC Food Pyramid				
	Q1	Q2	Q3	Q4	Q5
**N (frequency)**	2311	2311	2311	2311	2310
**SENC FP score **	59.9 (7.1)	72.1 (2.3)	79.4 (2.1)	87.5 (2.8)	101.5 (7.4)
**Age (years)**	34.7 (10.7)	34.7 (10.7)	34.7 (10.7)	34.7 (10.8)	34.8 (11.0)
**Female (%)**	72.8	73.0	72.9	72.9	73.3
**Body Mass Index (kg/m^2^)**	21.6 (2.0)	21.7 (1.9)	21.7 (1.9)	21.8 (1.9)	21.7 (1.9)
**Unemployed (%)**	6.8	6.4	6.2	6.5	5.0
**Married (%)**	43.3	43.8	42.5	43.4	41.5
**Living alone (%)**	7.1	6.2	6.3	6.8	8.5
**Special diet^a^ (%)**	4.6	5.4	5.2	6.3	8.6
**Snacking (%)**	34.5	36.1	32.1	32.7	28.6
**Smoking status (%)**					
**Never**	45.9	52.7	53.4	54.5	58.3
**Current**	29.6	24.2	21.4	20.6	16.1
**Years of education at university**	5.0 (1.4)	5.0 (1.5)	5.0 (1.5)	5.0 (1.5)	5.0 (1.5)
**Family history of obesity (%)**	17.0	19.5	19.9	21.1	24.3
**Physical activity (METs-h/week)**	19.9 (20.9)	20.7 (20.2)	23.3 (21.5)	24.2 (21.8)	30.0 (28.7)
**Television viewing (h/d)**	1.6 (1.2)	1.6 (1.2)	1.6 (1.2)	1.5 (1.2)	1.5 (1.2)
**Siesta >30 min (%)**	16.9	14.9	15.0	15.6	15.0
**Energy intake (kcal/d)**	2070 (625)	2261 (577)	2377 (578)	2493 (563)	2609 (556)
**Macronutrients (% E)**					
**Carbohydrate**	41.1 (8.0)	42.4 (6.9)	43.5 (6.8)	44.4 (6.8)	46.8 (6.6)
**Protein**	18.0 (4.1)	18.2 (3.1)	17.9 (2.9)	17.9 (2.9)	17.9 (2.8)
**Fat**	38.5 (7.0)	37.8 (6.3)	37.0 (6.3)	36.4 (6.3)	34.1 (6.3)
**SFA**	14.0 (3.6)	13.3 (2.9)	12.7 (2.8)	12.0 (2.8)	10.6 (2.7)
**MUFA**	16.1 (3.9)	15.9 (3.6)	15.9 (3.7)	15.9 (3.8)	15.1 (3.7)
**PUFA**	5.5 (1.9)	5.4 (1.6)	5.2 (1.5)	5.1 (1.4)	4.8 (1.3)
**SENC FP components (servings/d)**					
**Grains and byproducts**	1.5 (1.1)	1.8 (1.2)	2.0 (1.3)	2.1 (1.3)	2.2 (1.3)
**Whole-grain bread**	0.05 (0.2)	0.1 (0.3)	0.2 (0.4)	0.3 (0.6)	0.5 (0.8)
**Fruits**	1.5 (1.2)	2.0 (1.2)	2.6 (1.5)	3.4 (2.0)	4.8 (2.3)
**Vegetables**	1.3 (0.9)	1.8 (0.9)	2.1 (1.0)	2.6 (1.5)	3.5 (1.7)
**Dairy**	2.0 (1.7)	2.3 (1.4)	2.4 (1.4)	2.5 (1.5)	2.7 (1.5)
**Protein-rich foods^b^**	1.5 (0.7)	1.7 (0.6)	1.7 (0.6)	1.8 (0.6)	1.9 (0.6)
**Olive oil**	0.9 (1.0)	1.2 (1.1)	1.5 (1.3)	1.8 (1.4)	2.2 (1.5)
**Red and processed meat**	1.6 (0.8)	1.6 (0.8)	1.6 (0.8)	1.5 (0.8)	1.3 (0.7)
**Sweets and salty snacks**	4.4 (2.5)	4.3 (2.4)	4.2 (2.3)	4.0 (2.2)	3.4 (2.1)
**Alcoholic beverages**	0.5 (0.9)	0.4 (0.6)	0.4 (0.6)	03 (0.5)	0.3 (0.5)
**Water**	3.1 (2.6)	4.0 (2.6)	4.7 (2.4)	5.0 (2.4)	5.6 (2.3)
**Micronutrients (mg/d)**					
**Vitamin C**	162 (90.3)	210 (88.0)	250 (104)	318 (138)	429 (174)
**Vitamin D**	2.9 (2.1)	3.4 (2.5)	3.6 (2.2)	3.9 (2.3)	4.5 (2.7)
**Ca**	1021 (505)	1151 (424)	1224 (408)	1324 (437)	1442 (433)
**Na**	3680 (2258)	3932 (2368)	3996 (2230)	4008 (2039)	3893 (1848)
**K**	3578 (1166)	4176 (1023)	4607 (1111)	5248 (1373)	6264 (1570)
**Mg**	322 (99.2)	370 (87.1)	405 (91.3)	454 (105)	528 (119)
**Folate (μg/d)**	278 (111)	340 (107)	389 (118)	466 (158)	581 (179)
**Total dietary fiber (g/d)**	18.2 (8.0)	22.6 (6.9)	26.6 (8.1)	31.9 (10.1)	41.1 (12.4)
**Total alcohol intake (g/d)**	7.1 (10.7)	5.2 (7.3)	5.1 (6.7)	4.8 (6.3)	4.4 (5.8)

Mean and standard deviation (SD), or %

*Adjusted through inverse probability weighting

^a^ Special diets were mainly hypocaloric, lipid-lowering and low-sodium diets.

^b^ Lean meat (chicken, turkey, rabbit), fish & shellfish, eggs, legumes, nuts.

**Table 4:** Age- and sex-adjusted* baseline characteristics of participants according to tertiles (T) of adherence to SENC Hydration Pyramid (HP): The SUN Project, 1999–2015.

Variable	Adherence to SENC Hydration Pyramid		
	T1	T2	T3
**N (frequency)**	3852	3851	3851
**SENC HP score**	10.3 (1.2)	11.0 (0.9)	19.8 (2.4)
**SENC FP score **	87.8 (14.2)	77.3 (12.6)	75.3 (14.8)
**Age (years)**	34.7 (11.0)	34.7 (11.0)	34.8 (10.4)
**Female (%)**	72.9	72.9	72.9
**Body Mass Index (kg/m^2^)**	21.8 (1.9)	21.7 (1.9)	21.7 (2.0)
**Unemployed (%)**	6.0	6.9	5.8
**Married (%)**	41.9	43.9	43.1
**Living alone (%)**	7.3	6.8	7.1
**Special diet (%)**	6.0	5.1	6.6
**Snacking (%)**	32.8	34.1	32.1
**Smoking status (%)**			
**Never**	52.4	55.3	51.0
**Current**	22.3	21.3	23.9
**Years of education at university**	5.0 (1.5)	5.0 (1.5)	5.0 (1.5)
**Family history of obesity (%)**	21.7	18.9	20.3
**Physical activity (METs-h/week)**	26.9 (25.8)	21.8 (21.1)	21.4 (20.1)
**Television viewing (h/d)**	1.6 (1.2)	1.5 (1.1)	1.6 (1.2)
**Siesta >30 min (%)**	16.3	15.8	14.2
**Energy intake (kcal/d)**	2632 (558)	2288 (572)	2169 (576)
**SENC HP components (servings/d)**			
**Water**	5.8 (2.4)	3.8 (2.1)	3.9 (2.7)
**Low/non-caloric & non-nutritive**	1.1 (1.5)	0.7 (1.0)	1.4 (1.3)
**Caloric & nutritive**	3.1 (1.8)	2.2 (1.3)	1.3 (0.9)
**Calorically sweetened & non-nutritive**	0.4 (0.7)	0.3 (0.4)	0.3 (0.4)
**Water from foods**	7.4 (2.7)	5.2 (1.7)	5.2 (2.3)
**Macronutrients (% E)**			
**Carbohydrate**	45.1 (7.0)	43.1 (7.2)	42.7 (7.3)
**Protein**	18.2 (3.0)	17.9 (3.2)	17.9 (3.3)
**Fat**	35.4 (6.4)	37.4 (6.5)	37.5 (6.6)
**SFA**	11.9 (3.1)	13.0 (3.2)	12.7 (3.2)
**MUFA**	15.2 (3.6)	16.0 (3.7)	16.1 (3.8)
**PUFA**	4.9 (1.4)	5.3 (1.6)	5.4 (1.6)
**SENC FP components (servings/d)**			
**Grains and byproducts**	2.1 (1.3)	1.9 (1.3)	1.7 (1.2)
**Whole-grain bread**	0.3 (0.6)	0.2 (0.5)	0.2 (0.5)
**Fruits**	3.8 (2.4)	2.4 (1.6)	2.4 (1.8)
**Vegetables**	2.9 (1.7)	1.9 (1.0)	2.0 (1.3)
**Dairy**	3.1 (1.7)	2.2 (1.2)	1.8 (1.2)
**Protein sources**	1.9 (0.7)	1.7 (0.6)	1.6 (0.6)
**Olive oil**	1.7 (1.4)	1.4 (1.3)	1.4 (1.3)
**Red and processed meat**	1.6 (0.8)	1.5 (0.8)	1.5 (0.8)
**Sweets and salty snacks**	4.4 (2.5)	4.1 (2.3)	3.8 (2.2)
**Alcoholic beverages**	0.4 (0.6)	0.4 (0.6)	0.4 (0.7)
**Total dietary fiber (g/d)**	33.9 (13.2)	25.3 (9.9)	25.0 (11.3)
**Total alcohol intake (g/d)**	5.2 (7.5)	5.0 (7.5)	5.8 (8.2)

*Adjusted through inverse probability weighting

The contributions of different food groups to the SENC FP score are shown in Table 5. Fruits and vegetables, which together explained more than 50% of the variability, were the major contributors to the SENC FP variability among participants from the SUN cohort.

**Table 5:** Main sources of variability in the adherence to the SENC Food Pyramid (SFP) of participants from the SUN cohort.

SFP Components	*R*^2^	Change in *R*^2^
Vegetables	0.39	
Fruits	0.61	0.22
Protein-rich foods	0.72	0.11
Grains and byproducts	0.81	0.09
Water	0.87	0.06
Red and processed meat	0.90	0.03
Alcohol	0.93	0.03
Olive oil	0.95	0.03
Dairy products	0.98	0.02

HRs and 95% CIs for the association between the SENC FP and risk of overweight/obesity during follow-up are shown in Table 6. Results suggested a beneficial effect in the highest quintile of adherence to the SENC FP, which was associated with a modestly reduced risk of overweight/obesity (HR for the highest quintile compared with the lowest quintile: 0.78; 95% CI: 0.67–0.91; p-trend: 0.007), When we took advantage of repeated measurements by fitting Cox models with updated dietary values after 10 y of follow-up, the protective association remained significant (HR = 0.77; 95% CI: 0.67–0.89; p-trend: 0.003).

**Table 6:** Hazard ratios (HR) and 95% confidence intervals (CI) for incident overweight/obesity according to quintiles (Q) of adherence to the SENC Food Pyramid in the SUN Project.

	Adherence to the SENC Food Pyramid					
	Q1	Q2	Q3	Q4	Q5	*p* trend
**N**	2311	2311	2311	2311	2310	
**Cases**	545	484	468	453	370	
**Person-years**	22,991	22,929	23,243	22,115	21,934	
**Age- and sex-adjusted**	1 (ref.)	0.93 (0.82, 1.05)	0.94 (0.83, 1.06)	1.00 (0.88, 1.13)	0.84 (0.74, 0.97)	0.063
**Repeated measures**	1 (ref.)	0.91 (0.81, 1.03)	0.92 (0.81 1.04)	0.99 (0.87, 1.12)	0.82 (0.72, 0.93)	0.010
**Multivariable adjusted^a^**	1 (ref.)	0.88 (0.78, 1.00)	0.93 (0.82, 1.06)	0.93 (0.81, 1.07)	0.78 (0.67, 0.91)	0.007
**Repeated measures**	1 (ref.)	0.88 (0.78, 0.99)	0.92 (0.81, 1.04)	0.94 (0.83, 1.07)	0.77 (0.67, 0.89)	0.003

Age was used as the underlying time variable in all the models.

All the models were stratified by age groups and year of recruitment.

^a^ Additionally adjusted for baseline BMI (kg/m^2^, continuous), physical activity (METs-h/week, quartiles), hours of TV watching (quartiles), smoking status (current, never, former), marital status (single, married, other), highest level of education achieved (graduate, postgraduate), total energy intake (kcal/day, continuous), snacking between meals (yes, no), following a special diet at baseline (yes, no), family history of obesity (yes, no), hours of siesta (0, >0 – ≤0.5, >0.5). Robust standard errors were used.

Absolute average yearly weight change (g/y) decreased across quintiles of the SENC FP. In the multivariable adjusted model, participants in the highest quintile presented a yearly weight change of 145 g (95% CI: 53–238) lower than those in the lowest quintile (Table 7).

**Table 7:** Estimates (differences and 95% confidence intervals) for average yearly weight change (g/y) according to quintiles (Q) of adherence to the SENC Food Pyramid in the SUN Project.

	Adherence to the SENC Food Pyramid					
	Q1	Q2	Q3	Q4	Q5	*p* trend
**Age- and sex-adjusted differences**	0 (ref)	-26 (-111 to 59)	-84 (-169 to 1.80)	-135 (-221 to -49)	-158 (-244 to -72)	<0.001
**Multivariable-adjusted difference**^**a**^	0 (ref)	-20 (-105 to 65)	-70 (-157 to 17)	-122 (-211 to -34)	-145 (-238 to -53)	<0.001
**Absolute yearly weight change (g), adjusted mean**^**a**^	449 (387 to 512)	429 (369 to 490)	379 (319 to 439)	327 (266 to 386)	304 (241 to 366)	

^a^ Additionally adjusted for baseline BMI (kg/m^2^, continuous), physical activity (METs-h/week, quartiles), hours of TV watching (quartiles), smoking status (current, never, former), marital status (single, married, other), highest level of education achieved (graduate, postgraduate), total energy intake (kcal/day, continuous), snacking between meals (yes, no), following a special diet at baseline (yes, no), family history of obesity (yes, no), hours of siesta (0, >0 – ≤0.5, >0.5) and year of recruitment.

**Fig 2:**
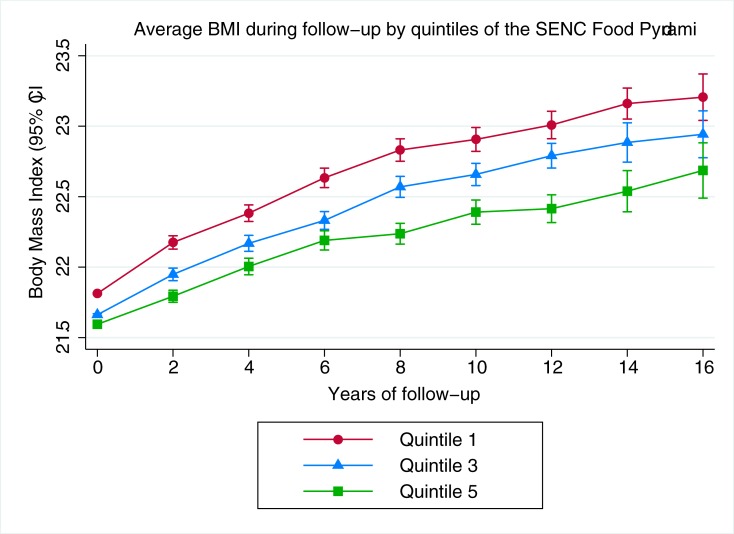
Generalized estimating equation model with average BMI (kg/m^2^, continuous) as the outcome during 16 y of follow-up according to extreme and median quintiles of the SENC (Spanish Society of Community Nutrition) Food Pyramid score. Multivariable model adjusted for sex, age, baseline BMI, physical activity, hours of TV watching, energy intake, smoking, marital status, level of education, sleeping siesta, snacking between meals, following a special diet, family history of obesity and year of recruitment.

Consistently, when generalized estimating equations were used to examine the relationship between quintiles of the SENC FP and biennially updated average BMI over time, we also observed modest differences in average BMI that were greater between extreme quintiles (Fig 2).

**Fig 3:**
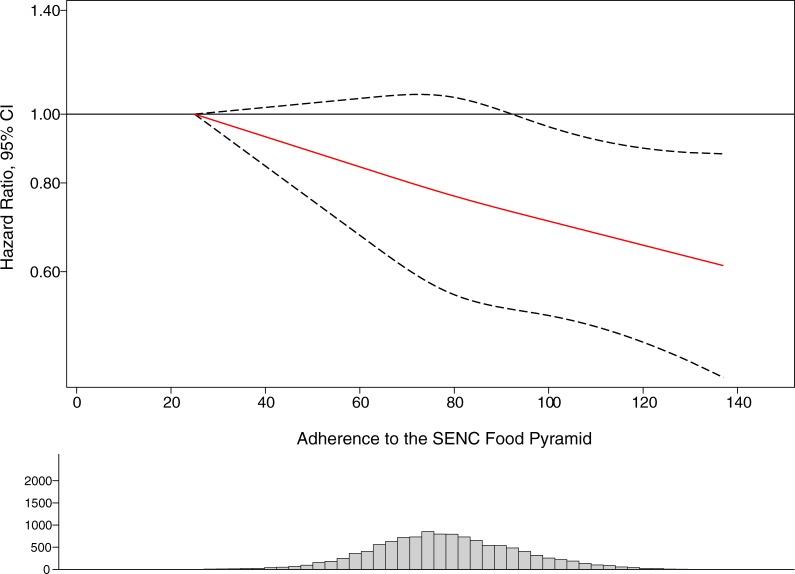
Restricted cubic splines of the association between the SENC (Spanish Society of Community Nutrition) Food Pyramid score and risk of overweight/obesity in the SUN cohort. Multivariable model adjusted for sex, age, baseline BMI, physical activity, hours of TV watching, smoking status, marital status, highest level of education achieved, total energy intake, snacking between meals, following a special diet at baseline, family history of obesity and hours of siesta. Age was used as the underlying time variable and model was stratified by age groups and year of recruitment.

Fig 3 represents the analysis using restricted cubic splines, which showed there was no deviation from linearity for the inverse SENC FP- overweight/obesity association (P for non-linearity = 0.171). Results suggest that moving from low/moderate to high (>80) adherence could be responsible for a reduction in the risk of overweight/obesity. However, only highest levels of adherence (>100) appear to be significantly protective.

In addition, we investigated the effect of a five-point increase in the individual SENC FP components on the risk of overweight/obesity (Table 8). The only single component significantly related to overweight/obesity risk was red and processed meat.

**Table 8:** Influence of a five-point increase in the single SENC Food Pyramid (FP) components on the risk of overweight/obesity.

SENC FP Component	Higher score reflects	Adjusted HR (95% CI)*
Vegetables	Higher consumption	1.01 (0.96, 1.05)
Fruits	Higher consumption	0.96 (0.92, 1.00)
Water	Higher consumption	1.04 (0.97, 1.11)
Grains and byproducts	Greater compliance with recommended consumption^a^	0.95 (0.89, 1.01)
Dairy	Greater compliance with recommended consumption^a^	1.01 (0.93, 1.10)
Protein-rich foods	Greater compliance with recommended consumption^a^	1.01 (0.97, 1.06)
Olive oil	Greater compliance with recommended consumption^a^	0.95 (0.87, 1.05)
**Red and processed meats**	**Lower consumption**	**0.81 (0.73, 0.91)**
Sweets, salty snacks and spreadable fats	Lower consumption	0.96 (0.86, 1.08)
Fermented alcoholic beverages	Lower consumption	0.99 (0.92, 1.07)

^a^ Too low as well as too high intakes had a penalizing effect on the score.

*Each component of the SENC FP was added into the model (adjusting for the other components). Adjusted for sex, baseline BMI, physical activity, hours of TV watching, smoking status, marital status, highest level of education achieved, total energy intake, snacking between meals, following a special diet at baseline, family history of obesity, hours of siesta. Stratified by age groups and year of recruitment.

There were no significant interaction effects between quintiles of adherence to the SENC FP score and sex, BMI, physical activity or age.

**Fig 4:**
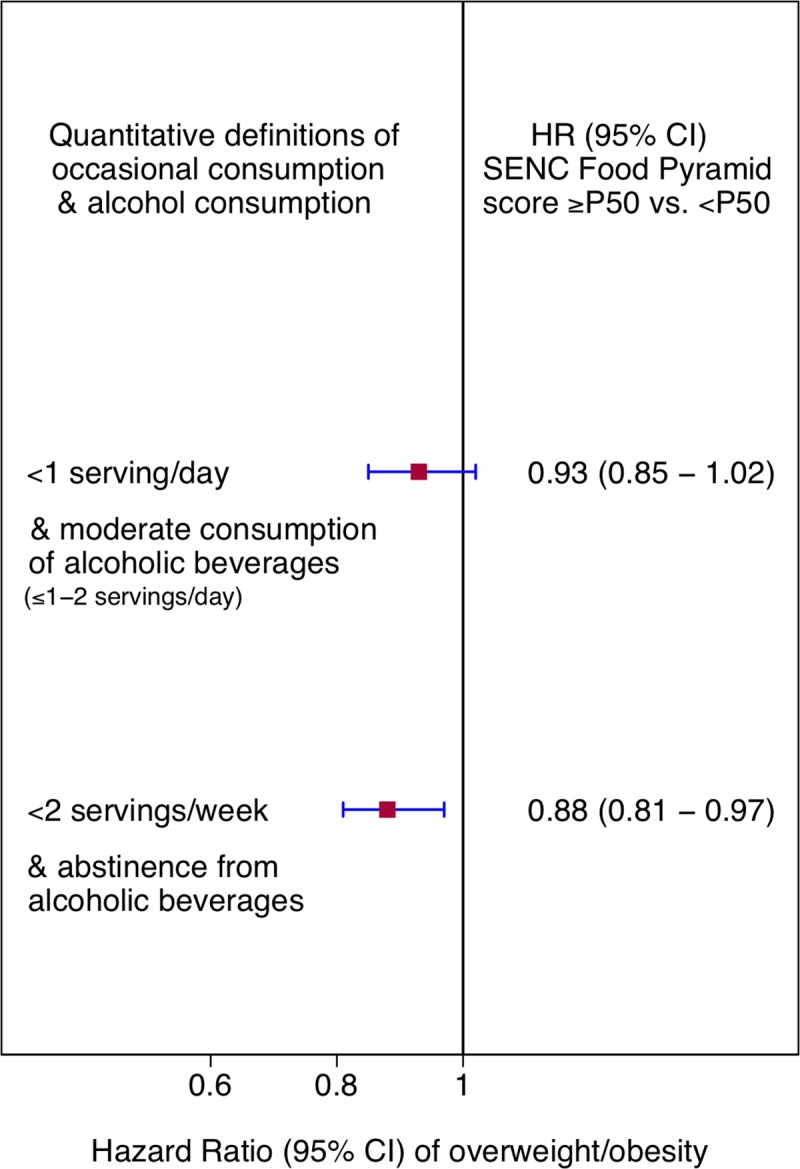
Multivariable-adjusted HRs for overweight/obesity according to adherence to the SENC Food Pyramid score (above vs. below the median [P50]) with different quantitative definitions for occasional food and alcohol consumption. The SENC FP does not provide quantitative guidance for food groups limited to an occasional consumption (red and processed meats, sweets, salty snacks and spreadable fats). We defined occasional consumption as <1 serving/day in our main analyses based on previous research. The SENC FP also includes the possibility of a moderate consumption of fermented alcoholic beverages (defined as 1–2 servings/day according to sex). Alternatively, we assessed the effect of the SENC FP on overweight/obesity risk defining occasional consumption as <2 servings/week and excluding the possibility of consuming alcohol.

An ancillary analysis aimed at assessing if a different interpretation of the frequency of occasional consumption and a different recommendation regarding alcohol consumption could lead to substantially different results is depicted in Fig 4. Results suggested that more explicit and restrictive guidance could lead to a greater protective effect. In fact, when SENC FP was analyzed as a dichotomous variable, defining occasional consumption as <1 serving/day and including a moderate consumption of alcoholic beverages (1–2 servings/day) resulted in a non-statistically significant association.

For the SENC HP, no consistent trends were found (Table 9). The multivariable-adjusted HR for participants in the highest tertile compared to those in the lowest tertile was 1.01 (95% CI 0.90–1.13; p- trend: 0.908).

**Table 9:** Hazard ratios (HR) and 95% confidence intervals (CI) for incident overweight/obesity according to tertiles (T) of adherence to the SENC Hydration Pyramid in the SUN Project.

	Adherence to the SENC Hydration Pyramid			
	T1	T2	T3	*p* trend
**N**	3852	3851	3851	
**Cases**	730	759	831	
**Person-years**	37,205	38,575	37,432	
**Age- and sex-adjusted**	1 (ref.)	0.92 (0.83, 1.01)	1.00 (0.91, 1.11)	0.289
**Repeated measures**	1 (ref.)	0.93 (0.84, 1.02)	1.00 (0.91, 1.11)	0.406
**Multivariable adjusted ^a^**	1 (ref.)	1.01 (0.90, 1.12)	1.01 (0.90, 1.13)	0.908
**Repeated measures**	1 (ref.)	1.00 (0.90, 1.11)	1.01 (0.90, 1.12)	0.899

Age was used as the underlying time variable in all the models.

All the models were stratified by age groups and year of recruitment.

^a^ Additionally adjusted for baseline BMI (kg/m^2^, continuous), physical activity (METs-h/week, quartiles), hours of TV watching (quartiles), smoking status (current, never, former), marital status (single, married, other), highest level of education achieved (graduate, postgraduate), total energy intake (kcal/day, continuous), snacking between meals (yes, no), following a special diet at baseline (yes, no), family history of obesity (yes, no), hours of siesta (0, >0 – ≤0.5, >0.5) and SENC FP score minus water (continuous).

Robust standard errors were used.

Finally, sensitivity analyses did not substantially change the main findings (Tables 10 and 11), reinforcing the robustness of our results.

**Table 10:** Sensitivity Analyses. Hazard Ratios (95% Confidence Intervals) of incident overweight/obesity for the fifth quintile (Q5) compared with the first quintile (Q1) of adherence to the SENC Food Pyramid.

	Cases	N	Q5 vs. Q1 (ref)	*p* trend
Overall	2320	11,554	0.78 (0.67, 0.91)	0.007
Energy limits: Percentiles 5–95	2301	11,637	0.81 (0.69, 0.94)	0.014
Excluding participants with no answer in >12 items out of 136 in the baseline FFQ	2116	10,613	0.81 (0.70, 0.95)	0.029
Excluding participants following a special diet at baseline	2122	10,875	0.77 (0.66, 0.91)	0.004
Including participants with weight change >10kg over the past 5 years before entering the study	2449	11,874	0.80 (0.60, 0.92)	0.015
Additionally adjusted for weight gain ≥3kg over the past 5 years before entering the cohort	2320	11,554	0.78 (0.68, 0.91)	0.008
Without adjusting for snacking between meals	2320	11,554	0.78 (0.67, 0.90)	0.005
Without adjusting for total energy intake	2320	11,554	0.83 (0.72, 0.96)	0.059
Excluding early cases of overweight/obesity (first 2 y)	1547	10,780	0.78 (0.65, 0.93)	0.015
Considering outcome as obesity (BMI ≥ 30 kg/m^2^)	759	15,489	0.75 (0.59, 0.96)	0.024
Truncating follow-up at 10 years	1884	5414	0.80 (0.69, 0.94)	0.049

Age was the underlying time variable in all models.

Adjusted for sex, baseline BMI, physical activity, hours of TV watching, smoking status, marital status, highest level of education achieved, total energy intake, snacking between meals, following a special diet at baseline, family history of obesity, hours of siesta. Stratified by age groups and year of recruitment.

**Table 11:** Sensitivity analyses. Hazard Ratios (95% Confidence Intervals) of incident overweight/obesity for the third tertile (T3) compared with the first tertile (T1) of adherence to the SENC Hydration Pyramid.

	Cases	N	T3 vs. T1 (ref)	*p* trend
Overall	2320	11,554	1.01 (0.90, 1.13)	0.908
Energy limits: Percentiles 5–95	2301	11,637	1.01 (0.91, 1.14)	0.762
Excluding participants with no answer in >12 items out of 136 in the baseline FFQ	2116	10,613	1.03 (0.91, 1.16)	0.501
Excluding participants following a special diet at baseline	2122	10,875	1.02 (0.91, 1.15)	0.760
Including participants with weight change >10kg over the past 5 years before entering the study	2449	11,874	0.99 (0.90, 1.09)	0.911
Additionally adjusted for weight gain ≥3kg over the past 5 years before entering the cohort	2320	11,554	1.01 (0.90, 1.13)	0.884
Without adjusting for snacking between meals	2320	11,554	1.01 (0.91, 1.13)	0.862
Without adjusting for total energy intake	2320	11,554	0.97 (0.87, 1.08)	0.658
Excluding early cases of overweight/obesity (first 2 y)	1547	10,780	0.96 (0.84, 1.10)	0.320
Considering outcome as obesity (BMI ≥ 30 kg/m^2^)	759	15,489	0.94 (0.77, 1.15)	0.508
Truncating follow-up at 10 years	1884	5414	1.02 (0.90, 1.15)	0.992

Age was the underlying time variable in all models.

Adjusted for sex, baseline BMI, physical activity, hours of TV watching, smoking status, marital status, highest level of education achieved, total energy intake, snacking between meals, following a special diet at baseline, family history of obesity, hours of siesta. Stratified by age groups and year of recruitment.

## Discussion

In the SUN cohort, better adherence to the SENC FP was modestly associated with a lower risk of overweight/obesity (HR = 0.78; 95% CI: 0.67–0.91; p-trend: 0.007), whereas no association was found for the SENC HP (HR = 1.01; 95% CI: 0.90–1.13; p-trend: 0.908).

Adherence to the SENC dietary guidelines and its association with obesity has been previously investigated in cross-sectional studies [24,25], which reported an inverse association between a higher SENC FP score and the risk of overweight/obesity. To our knowledge, the present study is the first prospective analysis to investigate this association and include the evaluation of the hydration guidelines (SENC HP), which do not seem to be independently associated with lower incidence of overweight/obesity. Taken together, results from our study suggest that recommendations from the SENC dietary guidelines are only partly effective in preventing long-term weight gain.

We acknowledge that dietary guidelines must be seen as part of a larger, intersectoral effort, involving improved public health policies and the promotion of healthy environments in order to be effective at obesity prevention. Nevertheless, they play an influential role in setting standards for nutrition and agricultural policies, and are the main reference for communities, health professionals, and government for promoting healthy eating. Ultimately, they have an influence on dietary choices, which represent a widely recognized target for preventing obesity and its associated comorbidities [34].

Although recent evidence suggests that greater dietary diversity (encouraged by dietary guidelines) is not necessarily beneficial to promote a healthy body weight [9,10], the inverse association between adherence to the SENC FP score and risk of overweight/obesity could be explained by the recommendations on physical activity and key food groups like fruits and vegetables. However, results suggest that guidelines could be considerably improved, and, as discussed subsequently, it could be argued that the lack of quantitative guidance and relevant updates are among the main challenges.

Dietary guidelines have always elicited controversy over the influential role of the food industry’s interests in their development, the focus on nutrients rather than foods, use of euphemisms and ambiguous terms (e.g., ‘moderate’ instead of ‘avoid’), conflicts of interest among committee members, and the process itself [22,23]. The 2015 Dietary Guidelines for Americans resolved some of these issues by developing the 2015 Dietary Guidelines Advisory Committee’s 571-page scientific report [35] and by focusing on healthy food patterns, which are the current state of the art in nutritional epidemiology [36,37]. The latest version of these guidelines was evaluated in relation to obesity risk obtaining favorable results [38].

Moreover, other countries like Australia have modified their dietary guidelines not only according to new evidence, but also addressing public concerns of those who considered that the message of the pyramid should not be to convey what not to eat. An updated version of their food pyramid in which foods that should be eaten in small amounts (i.e., those high in fats, sugar and salt) were replaced by healthy fats like olive oil was launched in 2013 [39], and research has shown that following these guidelines is linked to 30% lower risk of obesity [40]. Likewise, most recent Canada's 2019 plate-style Food Guide introduced some major changes: dairy have lost their prominent position and minimally processed plant-based foods have been prioritized. To ensure that the development of dietary guidance is free from conflict of interest, industry-commissioned reports were excluded from the review process. Importantly, they also acknowledged that healthy eating is a shared responsibility, encouraging governments to take action [41].

European countries like Sweden and Denmark have chosen to simplify their dietary guidelines and mainly focus on key foods to eat more or less of, rather than providing a comprehensive description of a daily diet [42]. This model has been recently adopted by the Public Health Agency of Catalonia, an autonomous community in Spain, which has published a new food guide seeking to clarify nutritional recommendations [43].

As a result of the nutrition transition and the increasing consumption and availability of highly processed foods, the 2014 Brazilian dietary guidelines [44] adopted the NOVA food classification, which is based on the nature, extent and purpose of food processing [45]. It has been suggested that this classification could be more nutritionally relevant than current food classifications based on botanical or animal origin, and could lead to improved and more meaningful dietary guidelines [46]. In the last few years, many studies have reported the non-salutary effects of ultra-processed food consumption [47], including a higher risk of overweight/obesity and mortality in the SUN cohort [48,49]. In addition, it is well documented that the processed food and beverage industry also contributes to the burden of disease through their aggressive marketing and attempts to shape government policy and public opinion in their favor [50]. Hence, national dietary guidelines issued recently in Latin American countries included explicit recommendations such as “avoid consumption of ultra-processed foods” and “be wary of food advertising and marketing” [51,52].

By contrast, the most widely used dietary guidelines in Spain, which are developed and authored by experts from the SENC, have changed very little over the last century. For instance, despite being the major source of carbohydrate intake in the Spanish population [53], grains and byproducts are still at the base of the FP. This situates other key carbohydrates sources like fruits and vegetables at a secondary level, at a time when their consumption is inadequate [54] and their role in prevention of chronic diseases is more than well-known. Furthermore, according to the SENC FP, energy intake from grains and byproducts should be adjusted based on the level of physical activity, which tends to be low in the general population [55]. On the other hand, the main novelties in the last updated version were the inclusion of factors like emotional status, energy balance, cooking methods, and nutritional supplements. Specific recommendations for physical activity and hydration were also added. Just like the aforementioned countries made timely changes, in the case of Spain it is not clear whether the updates were aligned with public health priorities. In fact, driving the attention to hydration, physical activity and energy balance has been recognized as one of the main ways in which some of the food industry biggest players reframe the debate about the causes of obesity and skew the evidence towards solutions that protect their financial interests [56–59].

In terms of message framing, it is also important to highlight that the advice to eat certain foods “in moderation” may lose its practical meaning in the absence of quantitative guidance and in the presence of contextual factors that are poorly aligned with moderate and healthy eating [8]. Nowadays, it is possible to choose from thousands of different discretionary or junk foods and portion sizes are increasingly big. In addition, confusing and ambiguous terminology is known to be further harnessed by some sections of the food industry who take advantage of it by lobbying against sensible regulations (i.e., tax sugary beverages) and using misleading marketing strategies [60]. It is also remarkable that, like other dietary guidelines, Spanish dietary guidelines include the possibility of a “moderate and responsible” consumption of alcoholic beverages. Although there are other relevant dimensions beyond the amount of alcohol consumed [61], it should not be a generalized option as the possible heart-related benefits of moderate alcohol consumption only outweigh increased risk of alcohol-related accidents in older people (60+) for which cardiovascular disease are the main cause of death [62]. On average, for the whole population, there is clear evidence that alcohol is a huge global health hazard and its consumption should not be encouraged in any way [63]. Findings from this study suggest that more restrictive recommendations regarding occasional food and alcohol consumption may be preferable in terms of obesity prevention. Similarly, a previous cross-sectional study evaluating the 2004 SENC dietary guidelines in relation to obesity showed that different definitions for the frequency of occasional consumption led to substantially different results. Moreover, the terms “moderate and responsible” place responsibility on individuals and ignore that individuals themselves have the least power over key factors driving consumption, such as taste, cost, convenience, or promotions. Noteworthy, the focus on personal responsibility can be used for policy inaction by favoring downstream interventions, which place high agency on individuals, rather than more effective, upstream, regulatory or fiscal strategies [64,65].

Another novelty of the latest SENC guidelines are the beverage recommendations represented in the HP. Nonetheless, our results regarding the SENC HP were not conclusive, as there was not a consistent trend relating HP adherence and risk of overweight/obesity. As highlighted in a review, there are other important aspects that warrant increased attention globally, such as the ecological impacts of diets and sociocultural factors including economic disparities and rapid dietary transitions toward ultraprocessed food consumption [66].

The present study has several limitations that should be taken into account. Dietary intake was self-reported through a FFQ so potential measurement error of the exposure, inherent to the methodology, could exist. However, the FFQ was previously validated [28,29] and this approach to assess habitual food consumption in large cohorts is in line with best practice recommendations [27]. Another methodologic limitation is that the FFQ used in our cohort did not gather information about consumption of whole-grain products (only bread), milk replacers, alcohol-free beer, tea nor sports drinks, which were considered in the SENC dietary guidelines. Other details like salt content of water and sugar content of milk, milk-based products, fruit juices and coffee were also unavailable. Moreover, we distinguished between coffee with or without sugar based on answers to “Do you add sugar to some beverages?”, but this question was not exclusive to coffee. Another potential limitation of our study is the self-reported nature of the outcome, which implies a potential source of information bias. Nevertheless, self-reported weight and BMI were previously validated in our cohort obtaining good correlation results [32]. In addition, our cohort is restricted to university graduates, so the sample is not representative of the Spanish population. However, this restriction allows us to control for socioeconomic status, and the homogeneity among participants reduces the likelihood of misclassification bias, reducing potential confounding and increasing internal validity. On the other hand, generalization should be based on biological plausibility rather than on statistical representativeness.

The strengths of our study include its prospective design, long follow-up period, relatively large sample size, high retention rate and ability to control for many potential sources of confounding. We were also able to minimize the possibility of reverse causation bias through exclusion of participants with baseline BMI ≥ 25 kg/m^2^, and reduce potential measurement errors with the use of repeated measurements of diet.

In conclusion, our results show that while adherence to the FP was associated with a lower overweight/obesity risk, the HP was not. Given that dietary guidelines are designed for overall well-being, further prospective studies examining other health outcomes are warranted. Ideally, dietary guidelines could then be strengthened accordingly in order to support the most appropriate nutritional education and food policies.
